# Personalized Transdiagnostic Cognitive Behavior Therapy With Midtreatment Stepped Care to Improve Mental Health Among University Students in Sweden: Feasibility Study for a Randomized Controlled Trial

**DOI:** 10.2196/68698

**Published:** 2026-01-15

**Authors:** Naira Topooco, Philip Lindner, Claes Andersson, Petra Lindfors, Olof Molander, Martin Kraepelien, Christopher Sundström, George Vlaescu, Gerhard Andersson, Marcus Bendtsen, Anne H Berman

**Affiliations:** 1 Department of Psychology Uppsala University Uppsala Sweden; 2 Centre for Psychiatry Research Department of Clinical Neuroscience Karolinska Institutet Stockholm Sweden; 3 Stockholm Health Care Services Region Stockholm Stockholm Sweden; 4 Department of Criminology Malmö University Malmö Sweden; 5 Department of Psychology Stockholm University Stockholm Sweden; 6 Department of Behavioural Sciences and Learning Linköping University Linköping, Östergötland Sweden; 7 Department of Biomedical and Clinical Sciences Linköping University Linköping Sweden; 8 Department of Health, Medicine and Caring Sciences Linköping University Linköping Sweden

**Keywords:** depression, anxiety, internet-based cognitive behavior therapy, university students, transdiagnostic, precision, eHealth, prevention, screening, WMH-ICS, World Mental Health International College Student

## Abstract

**Background:**

University students show a high prevalence of diverse mental health problems, requiring adaptable interventions to assist them in improving their mental health.

**Objective:**

This study aimed to evaluate the feasibility of transdiagnostic internet-delivered cognitive behavioral therapy (ICBT) for anxiety and depression in preparation for a randomized controlled trial. ICBT incorporated 2 innovative approaches to increase precision: user-steered content personalization and within-treatment adaptive modification based on early symptom trajectory.

**Methods:**

This single-group, open-label study was conducted online in Sweden in the autumn of 2021, recruiting from students who had completed the World Health Organization (WHO) World Mental Health International College Student (WMH-ICS) mental health survey. Participants were eligible if they scored 5-19 on the Patient Health Questionnaire-9 (PHQ-9), or ≥5 on the Generalized Anxiety Disorder-7 (GAD-7), or both. Participants completed an 8-week ICBT program with therapist support. They initially personalized their program by selecting a primary problem orientation, anxiety or depression, and choosing additional elective modules, and could consult their therapist regarding these choices. At midtreatment, stepped care was piloted, in which participants without symptom improvement were randomized to adaptive enhancement of therapist support or to continue treatment as before. The main feasibility outcomes included data on reach and uptake, intervention acceptability, stepped care procedures, and assessment retention up to 6 months. The GAD-7 and PHQ-9 were the primary outcome measures, with changes in scores calculated using mixed effects models.

**Results:**

Of 749 invited students, 55 (7%) completed the study screening, and 28 (4%) were included. The GAD-7 baseline score was 9.5 (SD 4.4), and the PHQ-9 baseline score was 11.2 (SD 5.2). Participants opened 6.2 (SD 2.2) out of the 8 treatment modules. The user-directed personalization yielded 27 unique treatment configurations across 28 participants. At week 4, 16/27 (59%) participants remaining in treatment were randomized in the stepped care procedure. Ratings on self-report measures showed acceptable to good therapeutic alliance and treatment satisfaction. Eleven participants reported increased stress associated with the treatment. Reductions in depression and anxiety symptoms were observed at postmeasurement and 6 months follow-up, with 43% attrition at those times.

**Conclusions:**

This pioneering study of personalized ICBT with adaptive change among university students demonstrated the overall feasibility of the treatment. To enhance the design of a future definitive trial, modifications are necessary to mitigate assessment attrition and reduce treatment-related stress.

**Trial Registration:**

ClinicalTrials.gov NCT05509660; https://clinicaltrials.gov/study/NCT05509660

## Introduction

### Background

Approximately one-third of university students report experiencing one or more probable mental disorders within the past 12 months, with mood and anxiety disorders being the most prevalent [1]. In Sweden, approximately 50% of young adults aged 18-29 years attend higher education [2], reporting higher rates of mental health problems compared to their nonacademic peers [3]. Despite a preference for psychological interventions over pharmacotherapy [4,5], the availability of such treatments often fails to meet demand [6]. This gap is further compounded by attitudinal barriers, including self-reliance and stigma, discouraging students from seeking mental health care [7,8]. Consequently, only approximately 25% of students with mental health disorders report receiving treatment in the past year [9]. Students request prompt and flexible access to mental health services, including after-hours support, reduced wait times, walk-in clinics, and online modalities [8]. Digital mental health interventions (DMHIs) have emerged as a promising strategy, garnering justifiable interest and acceptance among students [5,8,10]. In Sweden, the near-universal daily internet use among young adults [11] supports the application of DMHIs. Notably, internet-delivered cognitive behavioral therapy (ICBT), a low-threshold DMHI for mild to moderate mental health problems, has been integrated into Swedish health care [12] based on evidence demonstrating its efficacy in alleviating mental health symptoms when including brief therapist support [13-15]. Nonetheless, ICBT has not been specifically developed or deployed for the student population, nor has it been integrated into student health services, which in Sweden serve as auxiliary primary care facilities for students [16]. The evidence for ICBT among students also highlights the need for improved efficacy. A meta-analysis demonstrated that DMHIs using CBT yield larger effect sizes in anxiety and depression compared to alternative therapeutic modalities [17]; nonetheless, the overall effect sizes remain small. Another meta-analysis highlighted high attrition rates and low engagement among students using DMHIs, suggesting a potential misalignment between intervention design and user preferences [18]. A possible significant contributing factor is the substantial heterogeneity in mental health presentations, contrasted with the limited scope of DMHIs, including ICBT programs. Consequently, this research is increasingly focused on enhancing ICBT efficacy by integrating precision-targeted strategies into treatment.

### Treatment Personalization

Treatment personalization aims to optimize treatment precision through clinician-guided efforts of tailoring interventions to individual patient needs [19]. This approach is supported by substantial evidence that integrating patient preferences enhances mental health outcomes [20-22], including better outcomes and reduced dropout when patients receive their preferred choice of therapy [22]. In the realm of ICBT, evolving from static, disorder-specific protocols to transdiagnostic, personalized frameworks to better address prevalent comorbidities such as depression and anxiety represents a form of treatment personalization [23]. A meta-analysis reports moderate to large uncontrolled effect sizes for transdiagnostic ICBT in anxiety and depression outcomes compared to controls, also indicating that a transdiagnostic approach may outperform disorder-specific ICBT for depression [24]. Recent studies have taken personalization a step further, exploring patient-driven tailoring of ICBT that aligns with patient co-decision principles [25]. A Swedish primary care trial demonstrated that ICBT for anxiety, where participants selected modules and the desired modality of therapist support, yielded greater symptom reduction and perceived control compared to standardized ICBT [26]. A factorial trial evaluated a user-driven personalization approach to ICBT for depression, where participants selected 6-13 modules from a pool of 15. Compared to a therapist-prescribed approach (8 modules), the user-driven approach resulted in small but statistically significant reductions in depressive symptoms [27]. The evidence for transdiagnostic and personalized ICBT among university students is limited, with inconsistency in findings across 3 prior studies: one study demonstrated moderate to large effects relative to a waitlist control [28], another found no significant benefit over treatment as usual [29], and a third reported higher remission rates for ICBT with therapist support compared to unsupported ICBT and treatment as usual [30]. These discrepancies highlight the need for research to clarify the efficacy.

### Precision Medicine

Precision medicine aims to enhance treatment precision through data-driven, algorithmic, and quantitative decision-making [19]. Notably, clinical data acquired during treatment can be more predictive of outcomes than baseline measures [31,32], and using real-time monitoring of outcomes can improve clinical results [33]. In this context, precision medicine is particularly pertinent to stepped care models, in which initial low-intensity interventions are escalated based on clinical indicators of response. However, conventional stepped care approaches require completing an entire course of low-threshold, entry-level treatment before assessing the need for escalation. This methodology may delay necessary adjustments, potentially leading to patient frustration, demoralization, and associated risk of dropout. To address this limitation, earlier decision points within the initial treatment process have been proposed [34]. ICBT presents a suitable entry treatment in stepped care frameworks, and its typically embedded monitoring systems facilitate within-treatment adaptive modifications. A Swedish primary care trial demonstrated that monitoring early symptom trajectories during ICBT for insomnia and implementing adaptive changes in the treatment for patients who were indicated as nonresponders early in treatment significantly improved treatment outcomes as compared to no change [34]. The adaptive change involved enhanced, more personalized therapist guidance for the remainder of treatment, in line with evidence on the role of human support in DMHI efficacy [35]. Another trial compared coach-supported ICBT incorporating an adaptive step-up component to full-protocol telephone-administered CBT for depression. The study demonstrated the noninferiority of the adaptive ICBT approach, which involved participants who were nonresponders at early, mid, or late treatment stages, based on symptom severity, being escalated to telephone-administered CBT [36]. A study on youth anxiety further supports this approach, reporting self-guided ICBT augmented with therapist support for initial nonresponders to be noninferior to ICBT including therapist support from the outset [37]. Collectively, these findings suggest that adaptive allocation of therapist resources based on clinical indicators may enhance precision and outcomes in ICBT.

### Objectives of the Study

No research has examined within-treatment adaptive modifications in ICBT among university students. Similarly, investigations into user-driven personalization within this demographic remain limited. Notably, no studies have concurrently explored the integration of adaptive modifications and personalization in ICBT, despite their shared potential to enhance treatment efficacy through increased precision. From a theoretical standpoint, combining these approaches could yield synergistic effects, thereby advancing the clinical utility of ICBT.

We propose to evaluate a personalized, midtreatment stepped-care ICBT framework targeting anxiety and depression among university students. Treatment will be offered following mental health screening within university settings to ensure relevance and applicability for future systematic university initiatives. The trial will compare self-guided and therapist-supported ICBT with adaptive and personalized features against a waitlist control [38]. Before conducting a definitive randomized controlled trial, preliminary feasibility assessments are essential to identify potential challenges, refine methodologies, and optimize trial design [39-41]. This feasibility study was undertaken to evaluate the proposed intervention and the trial procedural framework. Given existing evidence supporting the feasibility of similar ICBT trial designs using the planned digital platform [27,42], a single-group design was chosen. This design focused on the most resource-intensive component, therapist-supported ICBT, and enabled the testing of all novel and core trial procedures and operational elements. Feasibility was assessed across multiple domains, including reach, uptake, engagement, treatment configuration, therapist support processes, participant acceptability (credibility, satisfaction, therapeutic alliance, and adverse events), and retention rates. Exploratory analyses of changes in primary depression and anxiety outcomes from baseline to follow-up were also conducted. No predefined feasibility benchmarks were established; instead, results were contextualized against current evidence from comparable procedures, DMHI frameworks, and student populations.

## Methods

### Study Design

This study used a 6-month single-arm feasibility trial in which all participants underwent ICBT treatment in an online setting at Uppsala University, Sweden. The study recruited students at 2 universities who had completed the Swedish branch of the World Mental Health Initiative Survey (WMH-ICS) epidemiological mental health screening [43] in the previous year. Recruitment occurred in October 2021, treatments were implemented from November to December 2021, and follow-up was conducted in April 2022. The study is reported in accordance with the CONSORT (Consolidated Standards of Reporting Trials) criteria applicable for nonrandomized feasibility studies [39] (Multimedia Appendix 1). The treatment is reported according to the TIDieR (Template for Intervention Description and Replication) [44] (Multimedia Appendix 2).

Eligibility inclusion criteria were ≥18 years, and meeting at least one of the following: score of 5-19 on the Patient Health Questionnaire-9 (PHQ-9; [45]), or score of ≥5 on the Generalized Anxiety Disorder-7 (GAD-7; [46]). Exclusion criteria were one or more of the following: ongoing other psychological treatment, antidepressant medication with a stable dose for less than 3 weeks, severe mental ill-health, or a score above 1 on the PHQ-9 suicidal ideation item in the screening.

### Procedures

Students received an email outlining the study objectives and intervention, along with a link to the consent and screening procedures. Eligible participants were subsequently invited to confirm their participation by completing a second pretreatment survey that included the secondary outcomes. Participants who completed this step proceeded to treatment initiation. At week 4 of 8 in treatment, participants who did not show symptomatic improvement were randomized to either adaptive treatment changes or to continue treatment as originally prescribed. Measures were collected at baseline, pretreatment, during treatment, posttreatment, and follow-up at 6, 12, and 24 months via email surveys, with 2 automated reminders. All but weekly measures were collected via Qualtrics (Qualtrics International Inc); weekly measures were collected in the study treatment platform [42]. Safety protocols included providing emergency contact information at treatment initiation, and monitoring and following up on symptom deterioration during treatments, with guidance to regular care resources as needed.

### Personalized Transdiagnostic CBT With Midtreatment Stepped Care

The treatment comprised a structured 8-module program delivered via a secure, web-based platform with a validated track record for administering DMHIs [42]. The treatment is rooted in CBT, emphasizing the interplay of cognitions and behaviors in symptom manifestation, with the objective of facilitating skill acquisition and application to improve these domains. Treatment modules were derived from ICBT protocols validated for depression and anxiety [47-49]. The investigators adapted the core anxiety and depression modules for a student population, informed by feedback from an MSc psychology student.

**Figure 1:**
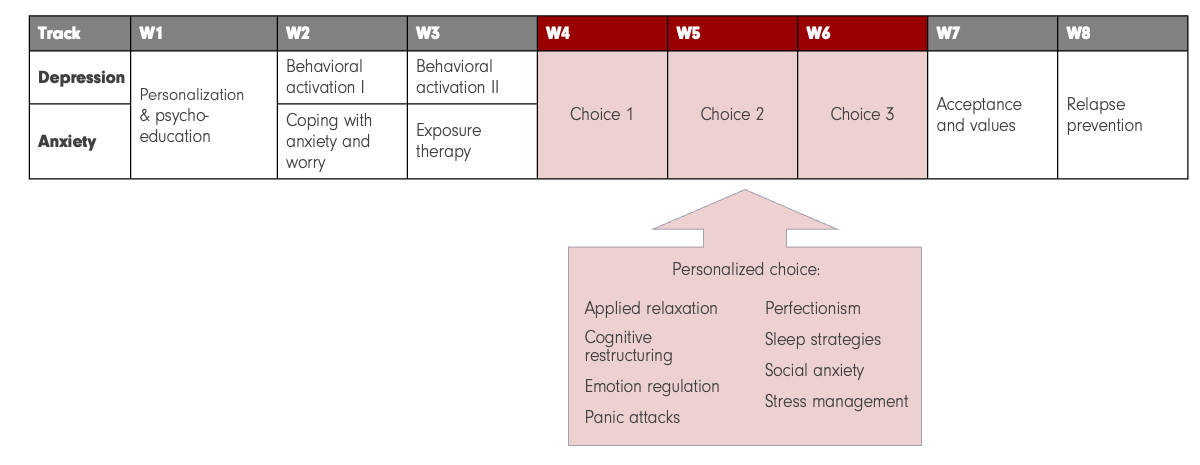
Treatment tracks and elective modules in personalized transdiagnostic 8-week internet-delivered cognitive behavioral therapy (ICBT).

Figure 1 shows the treatment outline. The treatment included brief written therapist support, user-steered personalization, as well as adaptive changes in therapist support based on early clinical indicators. Treatments were delivered individually and commenced with an introductory phone call with the assigned therapist, during which the treatment and administrative details were outlined and any questions resolved. All participants initiated treatment with a psychoeducational module focusing on the theoretical underpinnings of CBT for depression and anxiety. As part of this module, based on written explanations, participants selected a primary treatment focus, either anxiety or depression, that determined the content of modules 2 and 3. They also chose 3 additional elective modules from a pool of 8, which dictated modules 4 through 6. The final 2 modules were standardized across all participants and included values-based and acceptance strategies, and skills maintenance. Participants had the opportunity to consult with therapists about their selections and could request changes to the contents before access. Individual module plans were programmed to be pushed weekly. Starting in week 5 of treatment, adaptive change was implemented for participants who had not demonstrated symptom improvement. The adaptive change entailed modifying the therapist’s support in terms of intensity, guidance, and the modality of contact. Modifications could include adding telephone check-ins, setting reminders, or providing weekly suggestions, with the participant and therapist collaboratively determining adjustments. The adaptive framework followed principles for a previously validated ICBT framework for insomnia [34].

### Therapists, Treatment Guidelines, and Treatment Fidelity

Therapist support was implemented within the framework of the pilot version of a national advanced course in digital psychology for clinicians, taught by investigators (NT and AHB). This entailed the intended implementation model for the definitive trial. The therapists included 2 licensed CBT psychologists and 14 therapists in training: final-year MSc students enrolled in a 5-year clinical psychology program specializing in CBT. Therapists underwent general and treatment-specific preparatory theoretical and technical training in DMHI implementation. In the treatment phase, therapists in training underwent mandatory weekly supervision with CBT psychologists experienced in ICBT (MK and CS) and adhered to a detailed study-specific manual that included illustrative examples, weekly checklists for treatment fidelity, and guidelines for permissible adaptive modifications in therapist support. The therapists’ fidelity to treatment was monitored during clinical supervision, supplemented by compliance monitoring by a clinical practicum coordinator. Clinical supervisors participated in treatment planning meetings and had access to the treatment manual.

### Feasibility and Acceptability Outcomes

Feasibility outcomes included reach and uptake, defined as the proportion (%) who initiated the study screen, were eligible for, and initiated treatment. Treatment engagement was measured using platform metrics, including module openings, skill practice completions, and treatment configurations. The therapist’s time spent in treatments was estimated via self-reports. Participants evaluated the following acceptability measures: (1) credibility via the Credibility-Expectancy Scale (CEQ; range 3-30; higher scores indicate greater credibility [50]), (2) therapeutic alliance with the Working Alliance Inventory-Short Revised (range 12-84; higher scores denote stronger alliance [51]), (3) treatment satisfaction with the Client Satisfaction Questionnaire (range 0-32; higher scores reflect greater satisfaction [52]), and (4) negative effects with the Negative Effects Questionnaire-20 (NEQ-20; [53]). The NEQ-20 identifies 20 potential negative effects; for those endorsed, the user indicates whether attributed to treatment and rates the impact severity from 0 (not at all) to 4 (extremely). An open-ended item is included. The CEQ was administered at pretreatment; other measures at midtreatment (week 4) and posttreatment. Reasons for participant dropout were documented.

### Eligibility for Adaptive Change in Treatment

Participants’ eligibility rates for adaptive change in treatment were determined based on their scores on the PHQ-9 and GAD-7 in the first 3 weeks of treatment. Specifically, eligibility was assessed by comparing week-one scores to the average of scores obtained at weeks 2 and 3. Participants who exhibited no improvement or showed deterioration on either or both measures across this period were eligible for adaptive change, as were participants with missing data. Participants were randomized (1:1) to receive adaptive change or to continue treatment without change.

### Adherence to the Assessment Schedule

Participants’ adherence was assessed by the proportion of completed assessments weekly in treatment, posttreatment, and 6-month follow-up. In addition to acceptability measures and the primary outcomes, the assessment protocol included a comprehensive battery of secondary measures planned for the definitive trial. These measures and their corresponding assessment points are detailed in the protocol for the definitive trial [38]. Implementation between this study and the trial is aligned, except that the trial includes an additional midtreatment assessment point for the CEQ.

### Symptom Severity Outcomes (Planned Primary Outcomes)

Anxiety was assessed using the GAD-7 (scores ranging from 0 to 21; higher scores indicate greater severity). Published cutoff scores for anxiety levels are: mild (scores 5-9), moderate (10-14), and severe anxiety (15-21). Depression severity was assessed using the PHQ-9 (scores ranging from 0 to 27; higher scores denote more severe depression). Published cutoff scores for depression levels are mild (5-9), moderate (10-14), moderate to severe (15-19), and severe depression (20-27).

### Analyses

The achievable sample size was determined by the available pool of students who had completed the WMH-ICS screening [43]; assessing the uptake rate in this setting was a study objective. No formal sample size calculation was performed. Descriptive statistics were computed using frequencies, means, and percentages. Means for self-reported acceptability measures were calculated for valid cases. Estimates of therapist time omitted cases of 0 minutes recorded for participants who had dropped out. For the GAD-7 and the PHQ-9, mixed-effects models with random intercepts using the nlme package in R Studio (José C Pinheiro and Douglas M Bates) [54] estimated changes in measures from baseline to posttreatment and to 6-month follow-up. Participants were randomized to adaptive treatment using the randomization function in IBM SPSS Statistics (version 28). For the NEQ-20 measure, only adverse effects attributed to the treatment were reported, with the average number based on all available assessments. When a participant reported the same type of effect at 2 assessment points, it was recorded as a single effect. Alignment of baseline symptom severity and selected treatment focus of depression or anxiety was exploratively examined (aligned, or not aligned) based on categorizing participants into predominant symptom domains based on severity thresholds for the PHQ-9 and GAD-7 (eg, score of 5-9 on the PHQ-9 indicating mild severity). In cases when severity levels were equivalent across both measures, participants were also categorized in alignment. An error in the distribution of the NEQ-20 resulted in missing information for the cause and severity of sleep problems in 3 responses. An error in the randomization to adaptive treatment led to the exclusion of 5 eligible participants who had unchanged symptom severity scores or a < 2.5-point deterioration.

### Ethical Considerations

Participants provided written informed consent before screening. They received no monetary or other incentives for study participation. Ethical approval was provided by the Swedish Ethical Review Authority (ref ID 2021-03599). The study was retrospectively registered on August 22, 2022 (ClinicalTrials.gov NCT05509660).

## Results

### Treatment Reach and Uptake

**Figure 2:**
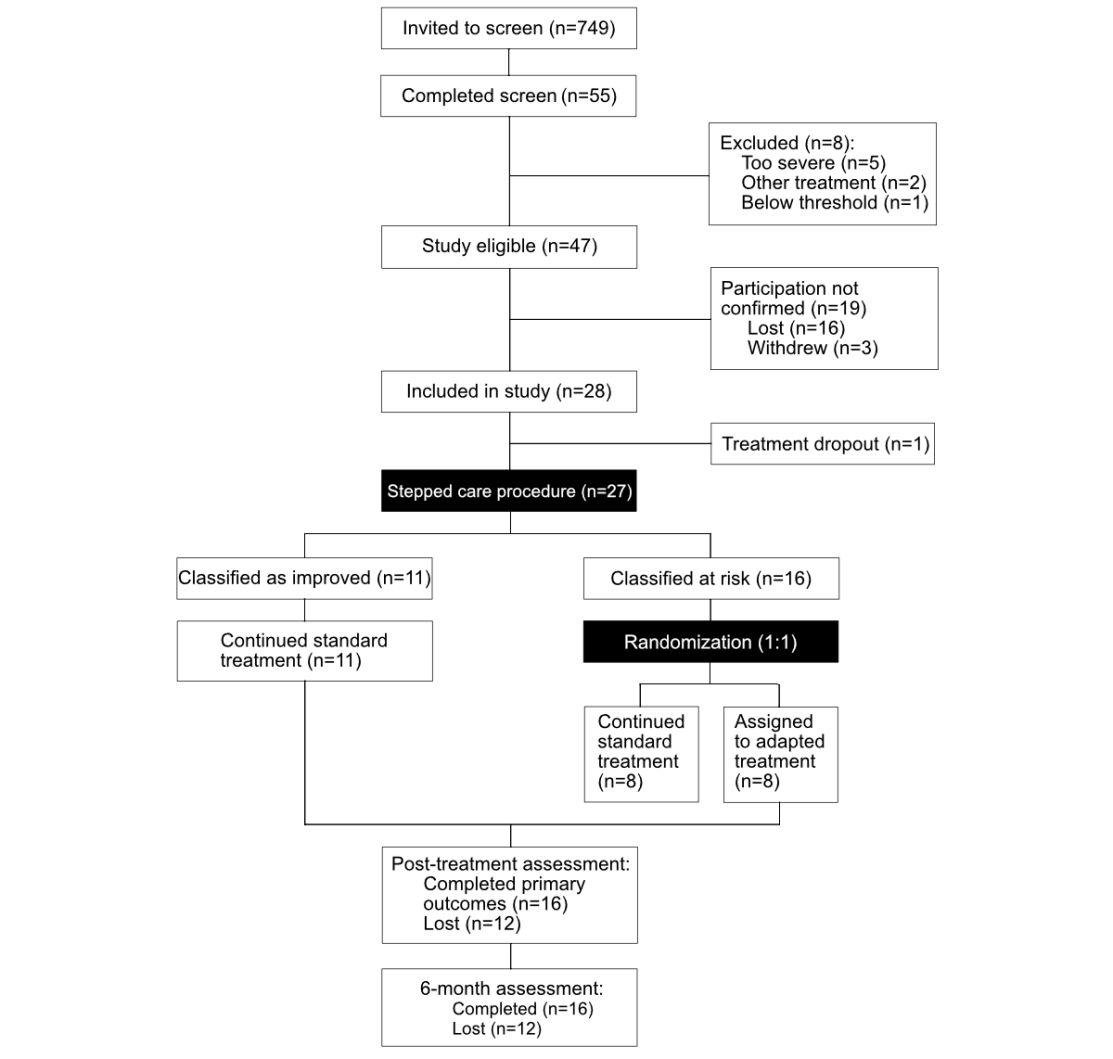
Flowchart of participants throughout the study.

Participant recruitment was conducted over a few weeks in alignment with therapist availability. Recruitment was discontinued once this predefined window closed. Figure 2 provides the study flowchart. Of 749 potential participants invited, 55 (7.3%) completed screening, with 47 (6.3%) meeting eligibility criteria. During the enrollment confirmation step, 19 (40.4%) withdrew or declined to continue, resulting in a final sample of 28 participants (59.6% of the eligible cohort). Two participants who did not fully meet the eligibility criteria were subsequently enrolled following a phone consultation with the principal investigator. Enrollment was permitted based on the study’s capacity to conduct treatment procedures and was deemed ethically justified considering the participants’ interest and potential benefits. These procedural exceptions do not apply to a definitive trial. The sample was predominantly female, 85.7% (24/28), and full-time students, 78.6% (22/28), with a mean age of 27.1 (SD 4.6) years. A minority identified as international students, 10.7% (3/28). The majority, 75% (21/28), exhibited both depressive and anxiety symptoms (ie, scores of ≥5). All participants initiated treatment.

### Treatment Engagement

Participants logged into the treatment platform a mean of 22.4 (SD 23.2; median 18, IQR 12-26.75) times, with one outlier exhibiting higher login frequency. Most participants (n=19, 67.9%) switched between modes in accessing treatment (computer, phone, and tablet), but the predominant mode of access was via computers, with a mean of 16.9 (SD 24.6) logins. Regarding treatment preference, 64.3% (n=18) selected ICBT for Depression (ICBT-DEP), and 35.7% (n=10) selected ICBT with an anxiety focus. On average, participants opened 6.2 (SD 2.2; range 2-8) modules out of 8. They completed 5 (SD 2.3; range 1-8) modules, defined as engaging in at least 1 homework exercise per module. ICBT-DEP participants opened 6.7 (SD 2; n=18) modules, whereas ICBT for anxiety participants opened 5.3 (SD 2.5; n=10) modules. Among participants eligible for adaptive change at midtreatment, those assigned to remain in the standard treatment mode opened 5 modules (n=8; SD 2.8; range 2-8), and those assigned to adaptive change opened 6.1 modules (n=8; SD 2.1; range 3-8). Participants not eligible for adaptive treatment change at midtreatment opened 7.4 (SD 1.2; n=11) modules. Tests for significant differences are not included due to small sample sizes.

Table 1 shows the distribution of elective module selections. Modules covering perfectionism (15/28, 54%), stress management (14/28, 50%), and cognitive restructuring (13/28, 46%) were most frequently chosen. Participants in ICBT-DEP predominantly selected the stress module, 56% (10/18). Regarding module prioritization, cognitive restructuring was most often selected for delivery first (8/28, 29%). Overall, the combination of treatment orientation, elective modules, and module order yielded 27 unique treatment configurations among 28 participants. Exploratory analysis showed that 76.9% (20/26) of participants selected a treatment track consistent with their relative baseline anxiety and depression levels. Participants who did not meet eligibility criteria were excluded from this categorization.

**Table 1:** Participants’ choice of elective content in transdiagnostic internet-delivered cognitive behavioral therapy (ICBT) treatment.

Module	Total^a^, n (%)	First priority^b^, n (%)
Perfectionism	15 (53.6)	4 (14.3)
Stress management	14 (50)	1 (3.6)
Cognitive restructuring	13 (46.4)	8 (28.6)
Emotion regulation	12 (42.9)	4 (14.3)
Social anxiety	10 (35.7)	4 (14.3)
Sleep strategies	9 (32.1)	4 (14.3)
Applied relaxation	7 (25)	2 (7.1)
Panic attacks	4 (14.3)	1 (3.6)

^a^Participants selecting the module as 1 of 3 elective options.

^b^Participants choosing the module to be delivered first among elective options.

### Credibility, Alliance, Satisfaction, and Negative Effects in Treatment

Participants rated a mean credibility score of 17.9 (SD 5.4; range 8-30; n=28) on the CEQ. Therapeutic alliance yielded WAI-S scores of 68.1 (SD 12.4; range 40-82; n=22) at midtreatment and 67.5 (SD 12.9; range 41-83; n=16) at posttreatment. Participants reported mean Client Satisfaction Questionnaire satisfaction scores of 22.6 (SD 5; range 11-29; n=22) at midtreatment and 23.5 (SD 6.1; range 9-31; n=15) at posttreatment. Among participants who completed assessments, 73% (16/22) reported negative effects attributed to the treatment. The mean number of effects was 2.9 (SD 2.9; range 0-9) out of 20, with “more stress” most frequently reported (n=11). Participants’ ratings of the severity of negative effects averaged 1.46 (SD 0.73; range 0-3) on a scale from 0 (not at all) to 4 (extremely), with most (12/16) rating impact below 2 (moderate). Free-text comments (n=7) included increased stress due to a demanding work-study schedule, feelings of gratitude or uncertainty toward the therapist, previous familiarity with the treatment content, and that the treatment did not adequately address how to handle real-life risk situations in daily life. Four participants ended treatment early, citing increased stress (n=3) or the treatment not suiting their problems (n=1). No adverse events were recorded in the study.

### Adaptive Change in Treatment and Therapist Time

At the time of the eligibility assessment for adaptive change in treatment, 16/27 (59.3%) of participants who remained in treatment became eligible; 7 out of 16 (43.8%) due to partially incomplete assessments. Correcting for participants mistakenly omitted (refer to “Analyses” section), 20/27 (74.1%) were eligible. In the second part of treatment, weeks 5-8, therapists spent on average 18.7 (SD 13.3; range 0-80) minutes per participant per week in the standard treatment and 22.4 (SD 13.3; range 5-60) minutes per participant per week in the adaptive treatment; a nonsignificant difference (t_93_=1.257; *P=*.21; 95% CI –9.58 to 2.15, mean difference -3.71).

### Adherence to the Assessment Schedule

During the treatment, self-reported assessments were available for between 89% (25/28) and 64% (18/28) of participants, depending on the week, with 177 out of 224 (79%) weekly measurements collected. At posttreatment, data for the PHQ-9 and GAD-7 were available for 16 out of 28 (57%) participants, and data for secondary outcomes were available for 15 out of 28 (54%) participants. At 6-month follow-up, data were available for 16 out of 28 (57%) participants.

### Primary Outcomes

In the total sample, there were reductions in overall symptom burden of depression and anxiety at posttreatment compared to baseline pretreatment levels, with similar reductions observed at 6-month follow-up. Multimedia Appendix 3 shows means, SDs, and exploratory Cohen *d* effects for the primary outcomes PHQ-9 and GAD-7 at pre- and posttreatment and at the 6-month follow-up.

## Discussion

### Principal Findings

This study represents the first investigation of personalized ICBT with midtreatment adaptive stepped care for university students experiencing depression and anxiety. The primary objective was to determine the feasibility of a definitive trial. Feasibility was demonstrated through key procedures: recruitment in an online university setting, delivery of the novel treatment features, and the implementation of therapist support within a therapist-in-training supervision model. Treatment acceptability was indicated by substantial engagement, personalization, and adequate ratings of therapeutic alliance and satisfaction. Exploratory examinations showed reductions in depression and anxiety following treatment, indicating that further investigation of efficacy is warranted. Identified challenges included assessment attrition and elevated stress levels associated with the treatment, necessitating modifications to improve these outcomes in a definitive trial.

The recruitment model of targeting students who had previously been screened for mental health issues resulted in a 7% screening completion rate, consistent with a meta-analysis of DMHIs recruiting college students in similar contexts [55]. The modest uptake likely reflects the broad invitation strategy, which did not tailor invitations based on previous symptomatology. In addition, students were approached approximately 1 year after they participated in the WMH-ICS screen. To improve the timeliness and potentially increase screening rates in the definitive trial, this interval will be shortened. Among students who completed the study screening, the eligibility rate was high, indicating successful engagement of students presenting within the targeted range of mild-to-moderate symptoms. A logistical challenge was the multistep enrollment process, which necessitated that eligible participants complete an additional survey to confirm participation and to be transferred onto the digital platform used for treatment; this step led to attrition. Previous research has identified key barriers to enrollment and initial engagement in DMHIs, including uncertainty about their usability and a perceived lack of value [56]. Given this, it is worthwhile to explore whether enhanced study communication about the CBT paradigm and potential benefits may increase initial interest and motivation to complete the enrollment procedure. Attrition ceased after enrollment, with all confirmed participants initiating treatment, indicating the acceptability of the treatment and the digital platform. By the time of a definitive trial, the eligible participant pool, comprising students with completed WMH-ICS surveys [43], is expected to have significantly increased, with over 10 universities anticipated to participate in the Swedish WMH-ICS. Accordingly, the projected uptake rates are acceptable as they stand.

Participants rated the therapeutic alliance and satisfaction as acceptable to good and engaged with an average of 78% (6.2/8) modules of treatment in a trial setting without monetary or other incentives for participation. Notably, self-prescribing treatment content led to 27 unique treatment configurations among 28 participants. The majority also selected treatment focus aligned with their relative baseline symptom severity of anxiety and depression. Adherence metrics confirm that, in general, participants engaged with all of their personalized content (modules 1-6). These findings indicate the treatment’s acceptability, and although important to note that assessing efficacy was not an objective in this study, the engagement levels are promising regarding the suggested dose-response relationship in behavioral interventions. Compared to previous reports for transdiagnostic ICBT with college students [28,29,57], the engagement levels appear favorable. A meta-analysis of DMHIs in student populations found that about 50% of studies achieved average completion rates of 70% or higher [58]. The integration of patient preferences and adaptive support mechanisms in the current treatment may positively affect treatment relevance and, thereby, engagement and adherence. This aligns with evidence that patient-centered treatment approaches can reduce dropout rates and improve clinical outcomes [20-22]. Participants’ content preferences related to baseline symptoms and outcomes will be investigated as part of a definitive trial.

The enrollment and eligibility rates derived from this study provide valuable estimates to inform therapist resources in a definitive trial. The eligibility criteria for the within-treatment stepped care procedure were highly inclusive, resulting in a high estimated maximum proportion of participants eligible for adaptive treatment modifications. However, a notable proportion of eligibility decisions were based on missing data, which may reflect participants’ perceived lack of benefit and could coincide with treatment dropout. While the procedures were designed to identify such cases, the accumulation of missing data before this step suggests that initiating the stepped care process even earlier in treatment may be advantageous to enhance treatment individualization and mitigate data attrition. This approach is supported by existing evidence indicating that early treatment response is predictive of clinical outcomes [31,32]. The findings for therapist support in this treatment support the feasibility of the implemented clinician-in-training model. The preparatory training and clinical supervision model used aligns with best-practice implementation of human support in DMHIs [59]. Although the workforce guiding within DMHIs is diverse, including personnel without clinical qualifications and peer support workers [59], this clinician-in-training model ensures the delivery of qualified, feedback-driven support. Past evaluations show that individuals participating in similar best-practice training report developing skills beneficial for their professional practice [60]. Therapists tended to allocate slightly more time to support in adaptive treatment than standard treatment, but variability was observed across cases. To preserve training standards, it is advisable to limit caseloads in the definitive trial, although increasing the caseload is feasible. During the feasibility assessment, therapist fidelity was monitored primarily through clinical supervision. For the upcoming definitive trial, monitoring will be expanded to include assessment of therapist adherence to the treatment manual checklists, such as whether feedback is sent on time and whether the content is accurate. Adaptive changes in treatments will be documented.

Challenges to treatment feasibility primarily stem from elevated stress levels among participants related to treatment, as identified through the systematic evaluation of treatment-related adverse effects. Although psychological treatments are generally effective, adverse effects, including increased stress, are not uncommon; approximately 1-2 out of 3 individuals undergoing psychological treatment report adverse outcomes, often involving symptom worsening [61]. In this study, participants rated the impact of stress and related effects primarily as “none” to “moderate,” suggesting limited overall influence; however, 3 participants discontinued treatment prematurely due to stress. Notably, at least half of the participants may have experienced pre-existing high stress levels, as evidenced by their selection of stress management content within their personalized treatment plans. Given that the treatment is largely self-managed and based on cognitive-behavioral principles involving self-directed skill practice, it inherently imposes internal pressures. Among university students, academic workload peaks and employment obligations are well-documented stressors [62]. Engagement in highly self-directed treatments, such as ICBT, amid these pressures likely increases the risk of exacerbated stress, particularly if participants perceive themselves as falling behind or struggling to find adequate time for engagement. Previous research reports that a “busy schedule” is a primary reason for dropout from transdiagnostic ICBT among college students [63], and a qualitative study reveals that some students experience frustration stemming from the perceived time-consuming and cognitively demanding nature of ICBT, and that perceived time constraints impede engagement [64]. These accumulating data underscore the need to explicitly address stress management in ICBT for university populations. To this end, a forthcoming trial will incorporate supplementary standardized and individualized procedures to mitigate and manage stress levels. These will include an expanded initial briefing for all participants, consisting of a standardized introductory message from the study team that will precede personalized messages regarding introductory phone calls and that concerns therapist support. This introductory communication will clarify the self-directed nature of the intervention, emphasize flexibility in engagement timing and methods, and specify a typical weekly time commitment of several hours. It will also communicate that the typical schedule involves completing 1 module per week, aligned with automated weekly delivery of treatment modules; however, it will explicitly advise participants to adapt the pace to their individual capacity if this is challenging, especially given the variability in module complexity. Furthermore, the briefing will explicitly note that there is a 2-week catch-up period after the final module, with all materials also available for download for continued or future use. Therapists’ training will also be revised to include additional guidance on the occurrence of stress among university students in ICBT, and therapists will be informed that participants’ selection of a stress management module may indicate increased stress risk. The protocol for therapists’ initial telephone consultation will be expanded to reiterate that, while modules are delivered weekly, circumstances (such as academic demands, illness, or additional work commitments) may necessitate acceptable pace adjustments. During treatment, the emphasis will be on tailoring the pace to individual needs rather than rigid adherence to module pacing. This aligns with the principles of personalized treatment. These measures aim to mitigate feelings of overwhelm or inappropriate pacing by improving participants’ preparedness and awareness of potential stressors, preventing potential misconceptions about participation requirements, and normalizing fluctuations in capacity and available time due to academic or personal circumstances. These measures will augment existing protocols for communicating with participants who express a desire to discontinue treatment, including emphasizing the option to continue treatment aligned with their capacity. Existing protocols also address risk management by continuously monitoring participants’ symptom severity and safety, through symptom tracking and communication with therapists and study staff, and by managing participants identified as at risk in accordance with established risk management protocols.

The study faced significant attrition rates in primary outcomes at both posttreatment and follow-up assessments, which threatens the validity of the findings by introducing bias and impairing interpretability. In a definitive trial, high attrition rates reduce statistical power and can lead to underestimation or overestimation of treatment effects, thereby compromising the efficacy evaluation [65]. Retention strategies in this study included 2 automated email reminders for assessments. Although less comprehensive than strategies involving telephone follow-ups or financial incentives, approaches often used in clinical trials, applying these strategies was not feasible in this study and will also not be feasible in a definitive trial due to funding constraints. Systematic reviews of trial retention strategies [66,67] recommend additional approaches, such as timely prompts, flexible data collection modalities, clear communication about the importance of follow-up, and guidelines for discussions about withdrawal. Despite the caveat that concerns about coercion may apply to some strategies, and despite the lack of high-quality evidence supporting the efficacy of recommended retention strategies [67], it remains essential to intensify retention efforts moving forward. Specifically, in a definitive trial, the retention strategy framework will be strengthened by increasing the frequency of reminders. Ethical approval will be sought to increase the number of reminders from 2 to 5 and to add SMS text messaging reminders, thereby enhancing participant engagement. In addition, the strategy will be expanded to include, as a last resort, an abbreviated assessment including only primary outcome measures for participants who remain unresponsive despite multiple reminders. This alternative assessment approach aims to reduce dropout rates and preserve essential data by reducing the assessment burden and focusing exclusively on primary outcomes. To account for the high attrition observed in this feasibility study, the sample size and statistical power calculations for the definitive trial will also be adjusted accordingly. This is especially pertinent given that the definitive trial will include long-term outcomes beyond 6 months, a period for which feasibility data on attrition are currently lacking. Increasing the sample size could partially offset attrition but may also introduce biases related to sample representativeness. Dropout analyses will be incorporated into the trial to identify potential biases and to strengthen the robustness of the findings.

The study experienced methodological shortcomings related to funding and resource constraints. The randomization errors highlight the need for more robust, multistep review processes to prevent similar issues in future trials. The protocol for the definitive trial will therefore be strengthened and safeguarded by involving additional team members in critical procedures. The study also raised concerns regarding retrospective registration and deviations from predefined eligibility criteria. The focus of feasibility studies is on testing procedures and interventions, and, relatedly, they are more flexible and less rigorous than trials [40]. However, retrospective registration and eligibility deviations are unacceptable in definitive trials and will not be applicable. Prospective registration for a definitive trial has been completed in accordance with established standards.

### Limitations

Study findings should be interpreted cautiously due to methodological limitations. While providing rich information on feasibility metrics to inform a definitive trial, the small sample size may have been insufficient for some feasibility parameters [68], and the missing data further limit conclusions. Allowing 2 participants who did not fully meet eligibility criteria constitutes a deviation that may bias results. Although the feasibility assessment was comprehensive and transparently reported, the absence of prospective registration is a limitation that undermines methodological rigor. The study did not predefine progression criteria for advancing to a definitive trial; instead, decisions relied on investigator judgment and existing literature on ICBT and university populations. While predefined feasibility criteria are recommended by guidelines, caution and flexibility are also advised to account for variability and avoid reliance on arbitrary thresholds that may distort feasibility assessments [39]. The feasibility evaluation followed the general principles of feasibility assessment, including necessary modifications before scaling. Ethical considerations concerned balancing potential benefits and risks. Another limitation is the lack of qualitative data on participants’ perspectives to clarify acceptability, especially regarding novel treatment features. Nevertheless, inclusion of validated feasibility measures, systematic retention monitoring, open-ended responses, and dropout reasons provided valuable contextual insights. While generalizability to real-world settings is not a primary aim of feasibility research, trial methods mirror those planned for a definitive trial and are also similar to those in Swedish primary health care for ICBT, where digital self-application and screening are increasingly adopted [12].

### Conclusion

This study represents the first evaluation of personalized, adaptive ICBT for the treatment of anxiety and depression in a university student population. Findings indicate the feasibility of this approach, supported by metrics of treatment engagement, personalization, and adequate acceptability ratings. However, challenges—notably poor assessment retention and treatment-related stress—were observed, necessitating the development of optimized frameworks to improve these outcomes in a definitive trial.

## Appendix

Multimedia Appendix 1 CONSORT 2010 checklist of items to include when reporting a pilot or feasibility trial.

Multimedia Appendix 2 The TIDieR (Template for Intervention Description and Replication) checklist.

Multimedia Appendix 3 Anxiety and depression outcomes.

## Abbreviations

CEQ: Credibility-Expectancy Scale
CONSORT: Consolidated Standards of Reporting Trials
DMHI: digital mental health intervention
GAD-7: General Anxiety Disorder-7
ICBT: internet-delivered cognitive behavior therapy
ICBT-DEP: ICBT for Depression
NEQ-20: Negative effects questionnaire-20
PHQ-9: Patient Health Questionnaire-9
TIDieR: Template for Intervention Description and Replication
WHO: World Health Organization
WMH-ICS: World Mental Health Initiative Survey

## Data Availability

The CBT treatment materials (in Swedish) are available upon request. The datasets analyzed during this study are available upon reasonable request.
