# Supplementary material for: Personalized Transdiagnostic Cognitive Behavior Therapy With Midtreatment Stepped Care to Improve Mental Health Among University Students in Sweden: Feasibility Study for a Randomized Controlled Trial
Source: JMIR Form Res. 2026 Jan 15;10:e68698. doi: 10.2196/68698 (PMC12856391; doi:10.2196/68698)
Supplement: Multimedia Appendix 3 [file formative_v10i1e68698_app3.docx]

| **Table S1.** Mean scores for planned anxiety and depression outcome measures^a^ | | | | | | | | | | | | | | | | |
| --- | --- | --- | --- | --- | --- | --- | --- | --- | --- | --- | --- | --- | --- | --- | --- | --- |
| Measure | Pre | | |  | Post | | |  | 6 months | | |  | Pre to post | | Pre to 6 months | |
|  | M | SD | N |  | M | SD | N |  | M | SD | N |  | *P* | Cohen’s d (95% CI) | *P* | Cohen’s d (95% CI) |
|  |  |  |  |  |  |  |  |  |  |  |  |  |  |  |  |  |
| **PHQ-9** |  |  |  |  |  |  |  |  |  |  |  |  |  |  |  |  |
|  | 11.2 | 5.2 | 28 |  | 5.7 | 3.3 | 16 |  | 5.0 | 3.9 | 16 |  | .0004 | 1.18  (0.51, 1.84) | .0001 | 1.28  (0.60, 1.95) |
| **GAD-7** |  |  |  |  |  |  |  |  |  |  |  |  |  |  |  |  |
|  | 9.5 | 4.4 | 28 |  | 4.8 | 2.9 | 16 |  | 4.2 | 2.8 | 16 |  | .0003 | 1.22  (0.55, 1.88) | .0001 | 1.36  (0.67, 2.03) |
| *Note.* Effect size distributions in Cohens *d* are included for clinical bench-marking purposes.  Abbreviations: M, Mean; SD, Standard deviation; N, number Pre, Baseline; Post, Post-treatment; 6 months = 6 month follow-up; PHQ-9, Patient Health Questionnaire-9; GAD-7, Generalized Anxiety Disorder-7. | | | | | | | | | | | | | | | | |
